# Foliate-Targeting Quantum Dots-*β*-Cyclodextrin Nanocarrier for Efficient Delivery of Unsymmetrical Bisacridines to Lung and Prostate Cancer Cells

**DOI:** 10.3390/ijms23031261

**Published:** 2022-01-23

**Authors:** Joanna Pilch, Patrycja Kowalik, Agata Kowalczyk, Piotr Bujak, Artur Kasprzak, Ewa Paluszkiewicz, Ewa Augustin, Anna M. Nowicka

**Affiliations:** 1 Faculty of Chemistry, Gdańsk University of Technology, Narutowicza Street 11/12, 80-233 Gdansk, Poland; ewa.paluszkiewicz@pg.edu.pl (E.P.); ewa.augustin@pg.edu.pl (E.A.); 2 Faculty of Chemistry, University of Warsaw, Pasteura Street 1, 02-093 Warsaw, Poland; patrycja.kowalik@student.uw.edu.pl (P.K.); akowalczyk@chem.uw.edu.pl (A.K.); 3 Faculty of Chemistry, Warsaw University of Technology, Noakowskiego Street 3, 00-664 Warsaw, Poland; piotrbujakchem@poczta.onet.pl (P.B.); akasprzak@ch.pw.edu.pl (A.K.)

**Keywords:** folic acid, unsymmetrical bisacridines, quantum dots, cellular uptake, mechanism of internalization, drug delivery

## Abstract

Targeted drug delivery by nanocarriers molecules can increase the efficiency of cancer treatment. One of the targeting ligands is folic acid (FA), which has a high affinity for the folic acid receptors, which are overexpressed in many cancers. Herein, we describe the preparation of the nanoconjugates containing quantum dots (QDs) and *β*-cyclodextrin (*β*-CD) with foliate-targeting properties for the delivery of anticancer compound C-2028. C-2028 was bound to the nanoconjugate via an inclusion complex with *β*-CD. The effect of using FA in QDs-*β*-CD(C-2028)-FA nanoconjugates on cytotoxicity, cellular uptake, and the mechanism of internalization in cancer (H460, Du-145, and LNCaP) and normal (MRC-5 and PNT1A) cells was investigated. The QDs-*β*-CD(C-2028)-FA were characterized using DLS (dynamic light scattering), ZP (zeta potential), quartz crystal microbalance with dissipation (QCM-D), and UV-vis spectroscopy. The conjugation of C-2028 with non-toxic QDs or QDs-*β*-CD-FA did not change the cytotoxicity of this compound. Confocal microscopy studies proved that the use of FA in nanoconjugates significantly increased the amount of delivered compound, especially to cancer cells. QD_green_-*β*-CD(C-2028)-FA enters the cells through multiple endocytosis pathways in different levels, depending on the cell line. To conclude, the use of FA is a good self-navigating molecule in the QDs platform for drug delivery to cancer cells.

## 1. Introduction

Significant progress has been made in effective cancer therapy. However, it is still the second cause of mortality in the world [1]. There are several problems with classical chemotherapy, including a lack of significant differences between cancer and normal cells, low bioavailability, high volume of drug distribution, as well as resistance to multiple drugs [2,3,4,5]. Therefore, to overcome these issues, new strategies for cancer treatment have been developed. One of them is based on the use of nanoparticles (NPs) as delivery platforms of anticancer drugs [6,7]. There are multiple types of NPs used in cancer therapy, including liposomes, micelles, polymeric and metal NPs, as well as quantum dots (QDs) [8]. These NPs can be used as multi-functional drug delivery platforms and additionally modified e.g., by conjugation with enzymes or antibodies [9]. Anticancer drugs can also be delivered with engineered NPs targeting surface proteins, which are overexpressed on the membrane of cancer cells in comparison to normal cells. Membrane proteins are involved in cancer progression, and they are the hallmark of a cancer cell. The overexpressed receptors on the cell membrane, including folic acid receptor (FR) [10], epidermal growth factor receptor (EGFR) [11], transferrin receptors (TfR1) [12], or G-protein-coupled receptors (GPCRs) [13], are becoming increasingly important in antitumor therapy [14]. The most intensely studied class of drug delivery is FRs and TfRs due to the high affinity of FA (folic acid) and Tf (transferrin), respectively [15,16]. Folic acid is a necessary nutrient for the synthesis of nucleotides in all living cells [17] and is taken up to the cells through the high-affinity folate receptors (FRs) via endocytosis [18]. Due to the low expression of FRs on normal cells and their overexpression on cancer cells, it is suggested that they can be used as navigating molecules in the selective delivery of chemotherapeutics to cancer cells [19]. It is expected that FR-targeted therapies can improve the efficiency of treating many types of cancer, which overexpress the FRs, including lung, kidney, brain, pancreas, stomach, prostate, and breast [20] but minimally expressed in normal tissues [15,20,21]. Navigating molecules-functionalized fluorescent agents, including folic acid- and transferrin-functionalized quantum dots can supply great assistance in surgery by tumor-specific fluorescence imaging [15] as well as drug targeting to cancer cells [22]. This strategy can allow for improving cancer therapy via enhancing the cytotoxicity and selectivity of drugs toward cancer cells while not affecting normal cells. Cyclodextrins (CDs) are amphiphilic cyclic oligosaccharides. The most common members of CDs are *α*-, *β*-, and *γ*-CDs. Due to their high stability, biocompatibility, and low immunogenicity, they have found various applications in the biotech, pharma, food, and cosmetic industry [23]. In recent years, CDs have become more frequently used materials in NPs-based drug delivery [24]. The use of CD in nanoconjugates has many benefits, including improved drug solubility and lipophilicity as well as the ability to overcome limitations of NPs such as low encapsulation efficiency and drug loading [6].

In our previous studies, we have applied quaternary QDs (Ag−In−Zn−S nanocrystals) as a platform transporting unsymmetrical bisacridines (UAs: C-2028 and C-2045) [25]. UAs are the newest group of potential anticancer drugs among acridine derivatives conducted in our department for many years. These compounds exhibited high cytotoxic and antitumor activity against many human tumors, including lung, pancreatic, prostate, and colon [26]. We showed that the efficient procedure for the synthesis of QD−UA hybrids successfully increases the cytotoxic activity of UAs in cancer H460 cells and has protective effects on normal MRC-5 and CCD 841 CoN cells [25]. We investigated also the influence of conjugation of UAs with QDs on the cellular uptake and the biological response in lung H460 and colon HCT116 cancer cells as well as normal MRC-5 and CCD 841 CoN cells [25,27].

In this paper, we report the effect of the use of folic acid as a navigating molecule in QDs-C-2028 nanoconjugates (QDs-*β*-CD(C-2028)-FA). C-2028 compound was selected for studies as the lead compound in a group of UAs with the strongest cytotoxic and antitumor activity. We showed the protocol of nanoconjugates synthesis, their quantitative and qualitative characteristics, as well as the results of their cytotoxic activity, cellular uptake, and mechanism of internalization in the lung (H460) and prostate (Du-145 and LNCaP) cancer cells as well as in MRC-5 and PNT1A normal cells.

## 2. Results and Discussion

### 2.1. Size and Stability of QDs-β-CD-FA-C-2028 Nanoconjugates

**Figure 1:**
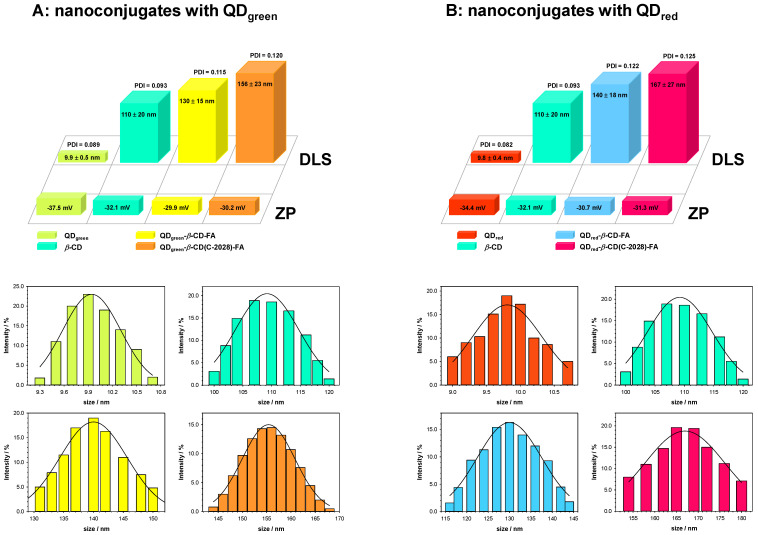
Hydrodynamic diameter, zeta potential, and normal distribution diagram of size for QDs, *β*-CD, QDs-*β*-CD-FA, and QDs-*β*-CD(C-2028)-FA nanoconjugates obtained in 0.02 M PBS buffer. Experimental conditions are *C*_QD-*β*-CD-FA_ = 5.0 × 10^−3^ mg × mL^−1^; *C*_C-2028_ = 1.0 µM; *C*_*β*-CD_ = 5.0 × 10^−3^ mg × mL^−1^.

One of the reasons for the high toxicity of existing anticancer drugs is their lack of selective action against cancer cells. At the same time, they show cytotoxic activity toward healthy cells (and tissues) [28]. For many years, anticancer compounds have been searched for selectively accumulating in neoplastic tissues. Despite extensive research, the search has not been successful so far. Recently, several research groups have been trying to achieve a selective effect of the drug by selecting the appropriate form of administration. High hopes are associated with drug forms containing active compounds conjugated with drug carriers in the form of nanoparticles, polymers, or liposomes [5]. It has been shown that some types of nanoparticles can selectively accumulate in neoplastic tissues. It was proved that the nanosized drug–carrier conjugates (<500 nm) easily penetrate tumor cells through their vasculature and accumulate inside omitting healthy tissues [29]. Thus, the studies were started with determining the size and stability of the QDs-*β*-CD-FA nanoconjugate and its complex with C-2028 (QDs-*β*-CD(C-2028)-FA). It is known that the presence of cyclodextrins favors the aggregation process [30,31]. Taking into account this fact, the DLS and ZP measurements were performed for two different QDs-*β*-CD(C-2028)-FA concentrations: 5.0 × 10^−3^ (the highest concentration used in cytotoxicity studies, Figure 1) and 1.0 mg × mL^−1^ (the final product of the synthesis, Figure S1 in the Supplementary Materials). The hydrodynamic diameter of QD_green_-*β*-CD-FA nanoconjugate after loading of C-2028 into the *β*-CD cavity increased by *ca*. 17% compared to the pure QD_s_-*β*-CD-FA nanoconjugate. It should be stressed that nanoconjugates with QD_red_ have a stronger tendency to accumulation compared to nanoconjugates with QD_green_. This tendency increases with concentration.

**Figure 2:**
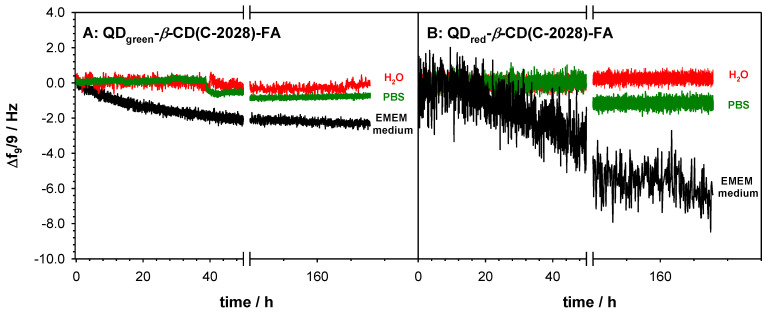
Frequency shifts of Au/QDs-*β*-CD(C-2028)-FA recorded in water, PBS buffer, and EMEM medium during 7 days. Experimental conditions: *C*_QD-*β*-CD(C-2028)-FA_ = 5.0 × 10^−3^ mg·mL^−1^.

It is well visible based on the values of the polydispersity index (PDI), which increases with subsequent modifications; see Figure S1. However, this increase is much higher in the case of nanoconjugates with QD_red_ and high concentration. It should be stressed that the highest concentration of QDs-*β*-CD(C-2028)-FA used in the experiments was 5.0 × 10^−3^ mg × mL^−1^ (which corresponds to the concentration of the C-2028 1.0 µM in the nanoconjugate). Taking into account the size of nanoconjugates for the highest used in experiments concentration (*ca.* 160 nm) and the cancer fenestrations formed during angiogenesis [32,33] (100–600 nm), the penetration of the cells by the nanoconjugate through the enhanced permeability and retention (EPR) effect is possible. Moreover, the introduction of the selective self-navigating molecule (FA) into the nanoconjugate should enhance the receptor-mediated endocytosis. The stability of the QDs-*β*-CD(C-2028)-FA nanoconjugate was defined based on the values of PDI and hydrodynamic diameter in three different environments: water, PBS buffer (pH 7.4), and EMEM medium. Stability measurements were performed for 7 days. Before each measurement, the solution was gently mixed. Based on the obtained results (Table 1), we can conclude that only the EMEM medium significantly affects the size of the nanoconjugate. The presence of large aggregates, ca. 1000 nm, was found only in this medium. The share of aggregates increased with time; initially, it was at *ca*. 5%, and after 7 days, it increased up to 20%. The aggregation process was the result of the rich composition of the EMEM medium. To get the information about the possible release of C-2028 from the QDs-*β*-CD(C-2028)-FA nanoconjugate, the gravimetric experiments were performed. The gold crystals were first modified with the nanoconjugates by placing the 100 μL droplet (5.0 × 10^−3^ mg × mL^−1^) and left to dry. Then, the crystals were placed in the chambers, and different media (water, PBS buffer, and EMEM medium) were introduced to the cell. The obtained frequency changes in the function of time are presented in Figure 2.

An increase in the frequency of quartz crystal vibrations was observed in none of the tested cases, which proves that the compound was not released from the conjugate. The obtained QCM-D results and ZP values, shown in Figure 1, proved that the QDs-*β*-CD(C-2028)-FA nanoconjugates are stable in the studied environments.

### 2.2. The Amount of C-2028 Accumulated in QDs-β-CD-FA Nanocarriers

**Figure 3:**
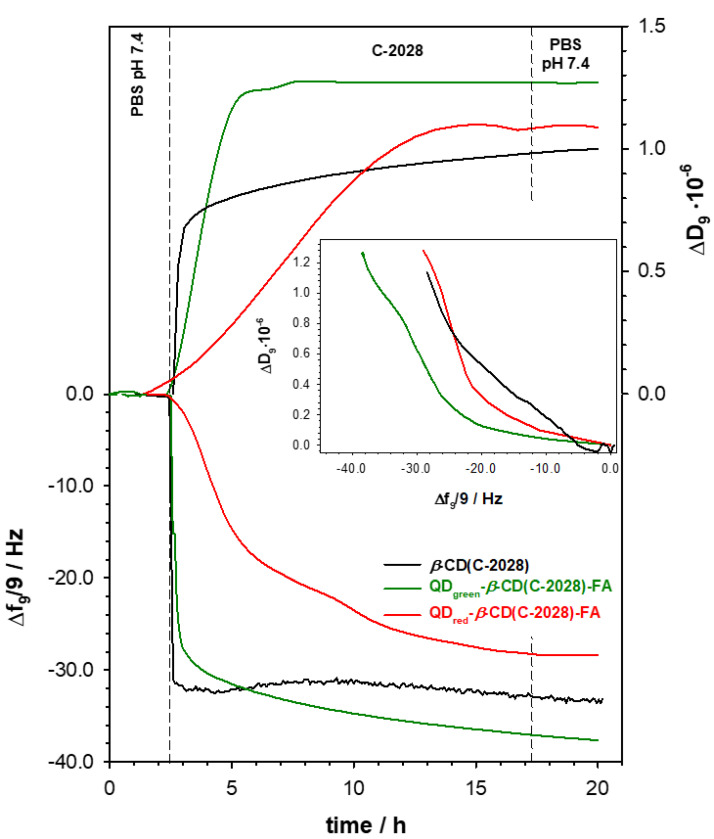
Typical QCM-D spectra of the shifts in frequency (Δ*f*) and dissipation factor (Δ*D*) were recorded during the interaction of C-2028 with Au/QD-*β*-CD-FA (red lines) and Au/*β*-CD (black lines). Inset: Δ*D* versus Δ*f* plots recorded during the formation of inclusion complex of C-2028 compound and *β*-CD present in the nanoconjugates at pH 7.4. Experimental conditions: 0.02 M PBS (pH 7.4); *C*_QD-*β*-CD-FA_ = 1.0 mg × mL^−1^; *C*_C-2028_ = 300 µM; *C*_*β*-CD_ = 1.0 mg × mL^−1^.

To determine the amount of C-2028 accumulated in the QDs-*β*-CD(C-2028)-FA nanoconjugates in the form of inclusion complex, the QCM-D measurements were performed using the 3rd to 13th overtones. The typical frequency (Δ*f*) and the dissipation factor (Δ*D*) changes, for one selected overtone, during loading of the C-2028 compound inside the *β*-CD cavity are presented in Figure 3. The gold quartz crystal was modified with QDs-*β*-CD-FA nanoconjugates outside of the QCM-D chamber. The 100 μL droplet containing the QDs-*β*-CD-FA nanoconjugates (1 mg × mL^−1^ in water) was placed on the surface of the Au sensor and left to dry in a desiccator. Such modified crystal (Au/QDs-*β*-CD-FA) was put in the QCM-D chamber and stabilized in 0.02 M PBS buffer at pH 7.4. After stabilization of the frequency dissipation factor, the C-2028 solution (100 μM) was added to the reaction chamber, and a drop in Δ*f* value and an increase in Δ*D* was observed. The decrease Δ*f* was due to the formation of an inclusion complex between *β*-CD and C-2028. After circa 10 h, the frequency shift reached a maximum of −37.0 Hz, and the solution in the chamber was changed on the pure 0.02 M PBS buffer (pH 7.4) to remove unbounded C-2028 molecules. The apparent mass increase relating to the step of the formation of inclusion complex (m_C-2028_ = Δ*f* × 17.7) with QDs-*β*-CD-FA nanoconjugates was 654.90 and 504.45 ng × cm^−2^ for QD_green_-*β*-CD-FA and QD_red_-*β*-CD-FA, respectively. The small changes of the Δ*f* during the washout step (PBS pH 7.4) in the case QD_green_-*β*-CD(C-2028)-FA was probably the consequence of the layer motion. This hypothesis is supported by the value of Δ*D/*Δ*f* ~3.4 × 10^−8^ Hz^−1^, which proved that the formed QD_green_-*β*-CD(C-2028)-FA nanoconjugate layer was not quite tight. It should be stressed that the QD_green_ nanocrystals, contrary to the QD_red_ nanocrystals, are characterized by two coordination spheres of ligands that stabilize zinc ions [25]. The first sphere contains ligands directly bound to the surface, and the second sphere includes free ligands that are in balance with the first sphere ligands. Probably this is the reason for such behavior. To prove that the presence of QD and selective self-navigating molecule (FA) in the nanoconjugate does not disturb the interaction of C-2028 with *β*-cyclodextrin on the way of inclusion complex, the control experiment with pure *β*-cyclodextrin was performed using QCM-D. Before the measurements, gold quartz crystal was modified with a *β*-CD layer formed during physical adsorption (C_*β*-CD_ = 1.0 mg × mL^−1^). As it is seen in Figure 3 (black lines), the frequency and the dissipation factor changes were very similar to those recorded during the interaction of C-2028 with the QD-*β*-CD-FA nanoconjugate. It means that the compound was introduced to the nanoconjugate only through the formation of the inclusion complexes with cyclodextrin.

Moreover, the dependences Δ*D* versus Δ*f* presented in the inset in Figure 3 confirmed that the nature of the inclusion complex did not change in the presence of QDs and FA in the nanoconjugates. Taking into account that the mass of QDs-*β*-CD-FA nanoconjugates modified the gold sensor and the mass relating to the step of the formation of inclusion complex between C-2028 and *β*-CD, the mass of the bisacridine derivative bounded to the 1 g of QDs-*β*-CD-FA nanocarriers was estimated and equaled 6.16 and 4.74 mg for QD_green_-*β*-CD-FA and QD_red_-*β*-CD-FA, respectively.

### 2.3. Cytotoxicity

The in vitro cytotoxicity of C-2028, *β*-CD(C-2028), *β*-CD (*β*-cyclodextrin), QDs (QD_green_ and QD_red_), QDs-*β*-CD-FA (QD_green_-*β*-CD-FA and QD_red_-*β*-CD-FA), and nanoconjugates: QDs-C-2028 (QD_green_-C-2028 and QD_red_-C-2028), as well as QDs-*β*-CD(C-2028)-FA (QD_green_-*β*-CD(C-2028)-FA and QD_red_-*β*-CD(C-2028)-FA) against tumor (H460, Du-145, LNCaP) and normal (MRC-5 and PNT1A) cell lines, were determined using the MTT assay. Results show that unbound QDs, *β*-CD, as well as QDs-*β*-CD-FA (without C-2028 compound) did not affect the growth of cancer and normal cells (Figure S2), which showed the excellent biocompatibility of these nanoparticles, indicating that they are good drug delivery platforms.

**Figure 4:**
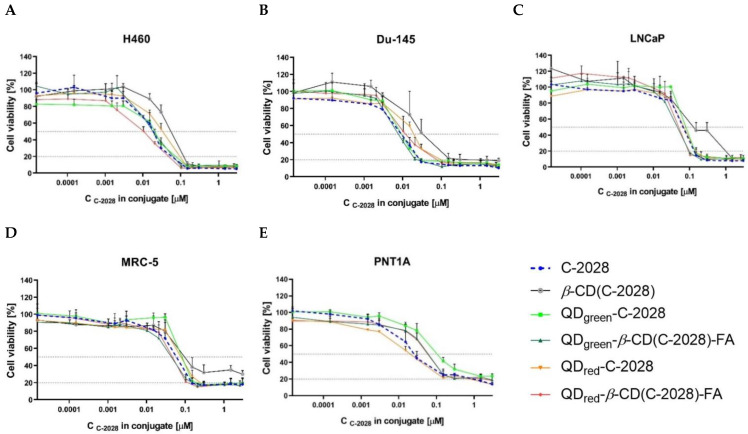
Growth inhibition of human cancer (**A**) H460, (**B**) Du-145, and (**C**) LNCaP cells as well as normal (**D**) MRC-5 and (**E**) PNT1A cells treated with increasing concentration of C-2028, *β*-CD(C-2028), QD_green_-C-2028, QD_green_-*β*-CD(C-2028)-FA, QD_red_-C-2028, and QD_red_-*β*-CD(C-2028)-FA following 72 h of incubation. Data are expressed as the mean ± of three independent experiments.

The data shown in Table 2 correspond to the concentration of C-2028 compound alone or conjugated with *β*-cyclodextrin (*β*-CD-C-2028), QDs (QDs-C-2028), and QDs-*β*-CD-FA (QDs-*β*-CD(C-2028)-FA) required to inhibit cell growth by 50% and 80% (IC_50_ and IC_80_, respectively). The growth inhibition curves of all these compounds, in all studied cell lines, are shown in Figure 4. The C-2028 compound exhibits high cytotoxic activity against the cancer cells of all the studied cell lines, especially toward H460 and Du-145 cells at IC_80_ value (0.035 and 0.024 µM, respectively). In the case of cancer LNCaP and normal MRC-5 cells, the IC_80_ value of the C-2028 compound was higher and corresponds to the values 0.133 and 0.138 µM, respectively. The studied compound displays the lowest cytotoxic activity against PNT1A normal cells (1.14 µM). Conjugation of C-2028 with *β*-CD in all of the studied cell lines significantly decreased the cytotoxicity of this compound. It is known that the conjugation of some anticancer drugs with cyclodextrin increases the bioavailability and reduces the toxicity of compounds, which is correlated with changes in the polarity and molecular dimensions of complexes [34]. The conjugation of C-2028 with QDs (QDs-C-2028 nanoconjugates) slightly decreases its cytotoxic activity (except QD_red_-C-2028 in the case of PNT1A normal cells). The use of folic acid in nanoconjugates (QDs-*β*-CD(C-2028)-FA) increases the cytotoxic activity of C-2028 compared to QDs-C-2028 in all of the studied cell lines. In normal cells, the small protective effect after conjugation C-2028 compound with QDs or QDs-*β*-CD-FA was observed only at an IC_50_ value. This effect was higher for nanoconjugates with QDs.

### 2.4. Cellular Uptake

**Figure 5:**
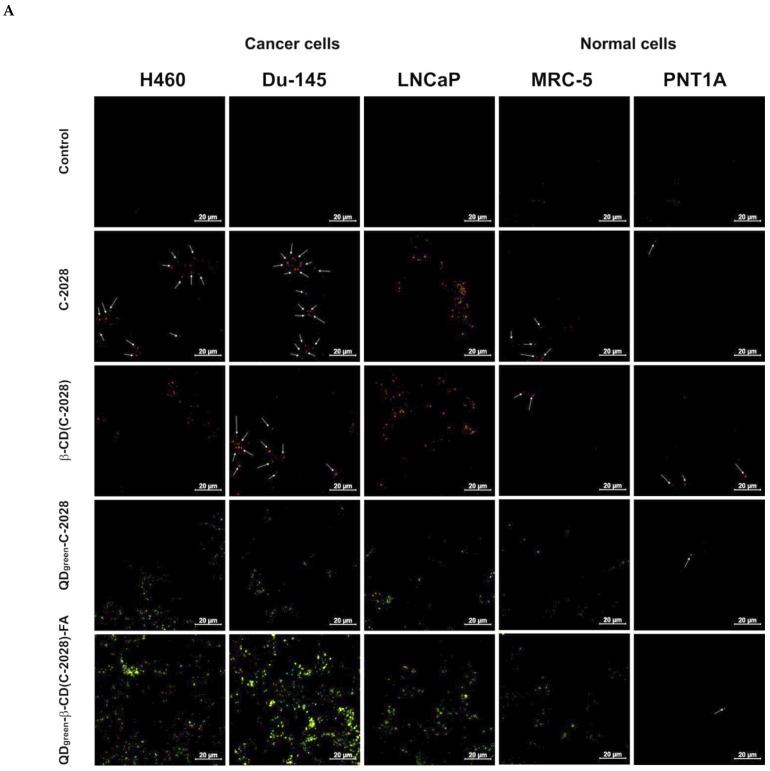

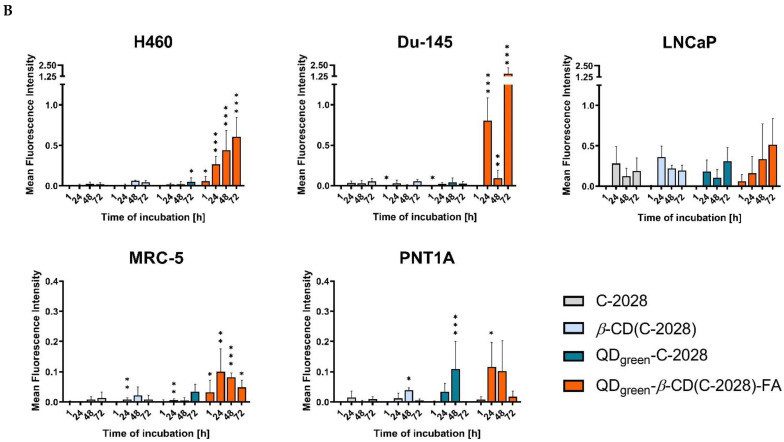
(**A**) Intracellular fluorescence images of C-2028, *β*-CD(C-2028), QD_green_-C-2028, and QD_green_-*β*-CD(C-2028)-FA by CLSM after 72 h of incubation in cancer: H460, Du-145, and LNCaP cells as well as in normal MRC-5 and PNT1A cells. C-2028 and QD_green_ are shown in orange and green, respectively. The scale bar is 20 μm. (**B**) Cellular uptake of C-2028, *β*-CD(C-2028), QD_green_-C-2028, and QD_green_-*β*-CD(C-2028)-FA nanoconjugates into studied cells for the time indicated and analyzed by CLSM. Mean Fluorescence Intensity (MFI) values of C-2028 and its nanoconjugates were determined using ImageJ software. Data are expressed as the mean ± SD, *n* = 3. * *p* < 0.05, ** *p* < 0.01, *** *p* < 0.001—statistically significant differences between the MFI of C-2028 in the cells incubated with C-2028 alone and its nanoconjugates in the cells (Student’s *t*-test).

Many factors, including particle size, shape, surface properties, time of incubation, and presence of navigating molecules may influence the cellular uptake of NPs and their nanoconjugates with drugs into cells [35]. Here, we studied the influence of navigating molecules (folic acid) and incubation time (1, 24, 48, and 72 h). Based on our previous experience with cellular uptake studies [27], we selected for further experiments nanoconjugates with QD_green_. The amount of the delivered C-2028 compound to cancer cells was higher in the case of the use of QD_green_ as a drug delivery platform compared to QD_red_. To evaluate the influence of FA in nanoconjugates on cellular uptake, Confocal Laser Scanning Microscopy (CLSM) was used. The time-dependent cellular uptake of QD_green_, *β*-CD, QD_green_-*β*-CD-FA, C-2028, *β*-CD-C-2028, QD_green_-C-2028, and QD_green_-*β*-CD(C-2028)-FA nanoconjugates were selected to further studies with cancer H460, Du-145, and LNCaP, as well as normal MRC-5 and PNT1A cells. Based on the fluorescence properties of these compounds, green and orange fluorescence are representative for QD_green_ and C-2028, respectively. As shown in Figure S3 (in the Supplementary Materials), the signals from unbound QD_green_ and QD_green_-*β*-CD-FA (without C-2028 compound) in all of the studied cell lines were at the limit of detectability and did not change with the prolongation of incubation in all cell lines. The signals from *β*-CD were not detected (Figure S3). CLSM study shows that the C-2028 alone enters cells, starting with 1 h of incubation (Figure 5 and Figure S4A–E in the Supplementary Materials). This tendency was stronger in the case of cancer cells. The full time-dependent cellular uptake of C-2028, *β*-CD(C-2028), QD_green_-C-2028, and QD_green_-*β*-CD(C-2028)-FA nanoconjugates to all of the studied cell lines is presented in Figure S4A–E in the Supplementary Materials. The conjugation of *β*-CD with C-2028 (*β*-CD-C-2028) did not affect the cellular uptake of this compound in cancer cells. In contrast, in normal cells, *β*-CD(C-2028) enters the cells more effectively than C-2028 alone. The conjugation of C-2028 with QD_green_ (QD_green_-C-2028) increases the number of delivered compounds. The use of navigating molecule (FA) in nanoconjugates (QD_green_-*β*-CD(C-2028)-FA) significantly improved the cellular uptake in cancer H460 and Du-145 cells. Importantly, this effect was stronger in lung and prostate tumors (H460 and Du-145), compared to normal cells (MRC-5 and PNT1A). In the case of prostate cancer LNCaP cells, no significant changes in the cellular uptake of nanoconjugates QD_green_-C-2028 as well as QD_green_-*β*-CD(C-2028)-FA (vs. C-2028 alone) were observed, which can be associated with negative folate receptors in this cell line [36]. Interestingly, the Du-145 cell line has a relatively low expression of FR too, but in this cell line, we observed a significantly increased cellular uptake of nanoconjugates with FA (Figure 5 and Figure S4B in the Supplementary Materials). That suggests that not only FR at the membrane of the cells plays an important role in the cellular uptake of QD_green_-*β*-CD(C-2028)-FA nanoconjugate. These cell lines have a different expression of other receptors on the cell membrane e.g., androgen receptor (AR), which are negative for Du-145 and PNT1A and positive for LNCaP, respectively [37,38]. Moreover, in cancer H460 and Du-145 cells, more QD_green_-*β*-CD(C-2028)-FA nanoconjugates were accumulated following prolonged incubation time in contrast to normal MRC-5 and PNT1A cells. In these cells, the highest mean fluorescence intensity (MFI) of QD_green_-*β*-CD(C-2028)-FA was observed following 24 h of incubation and, after this time, the MFI of the nanoconjugates started to decrease. The low expression of FRs in normal cells may be responsible for the weak cellular uptake of nanoconjugates with FA [15,20,21].

### 2.5. Internalization Study

It is well known that foreign materials e.g., nanoparticles, can be internalized to the cells via endocytosis—phagocytosis or pinocytosis (CME—clathrin-mediated endocytosis, CavME—caveolae-mediated endocytosis, and MP—macropinocytosis) [39,40]. The cellular fate of internalized materials can be different, depending on the type of endocytosis. For example, clathrin-mediated endocytosis directs the materials to lysosomes/acidic endosomal compartments [41]. This phenomenon can be used in the drug delivery approach, where hydrolytic enzymes in lysosomes degraded nanoconjugates and released chemotherapeutics [42]. In our previous studies, we showed that QDs−UAs nanoconjugates, after their internalization, localized in lysosomes, where UAs released from the nanoconjugates [25]. It is known that the endocytotic pathway via a folic acid receptor (FR) is mediated by two proteins, clathrin and caveolin [43]. CLMS study shows that cellular uptake of QD_green_-*β*-CD(C-2028)-FA nanoconjugate was delivered with different efficiencies, depending on cell lines. Therefore, the next step of our study was to investigate the mechanism of the internalization of this nanoconjugate. We employed the most popular inhibitors of different pathways of endocytosis in non-toxic concentration, including an inhibitor of the energy-dependent process (4 °C), an inhibitor of MP and/or phagocytosis (Cytochalasin D and Amiloride), an inhibitor CME (Dynasore and Pitstop 2), and an inhibitor of CavME (Filipin III) [44]. First, all of the studied cells (H460, Du-145, LNCaP, MRC-5, and PNT1A) were exposed to these inhibitors for 30 min and next incubated with QD_green_-*β*-CD(C-2028)-FA nanoconjugate for 4 h (in the presence of inhibitors).

**Figure 6:**
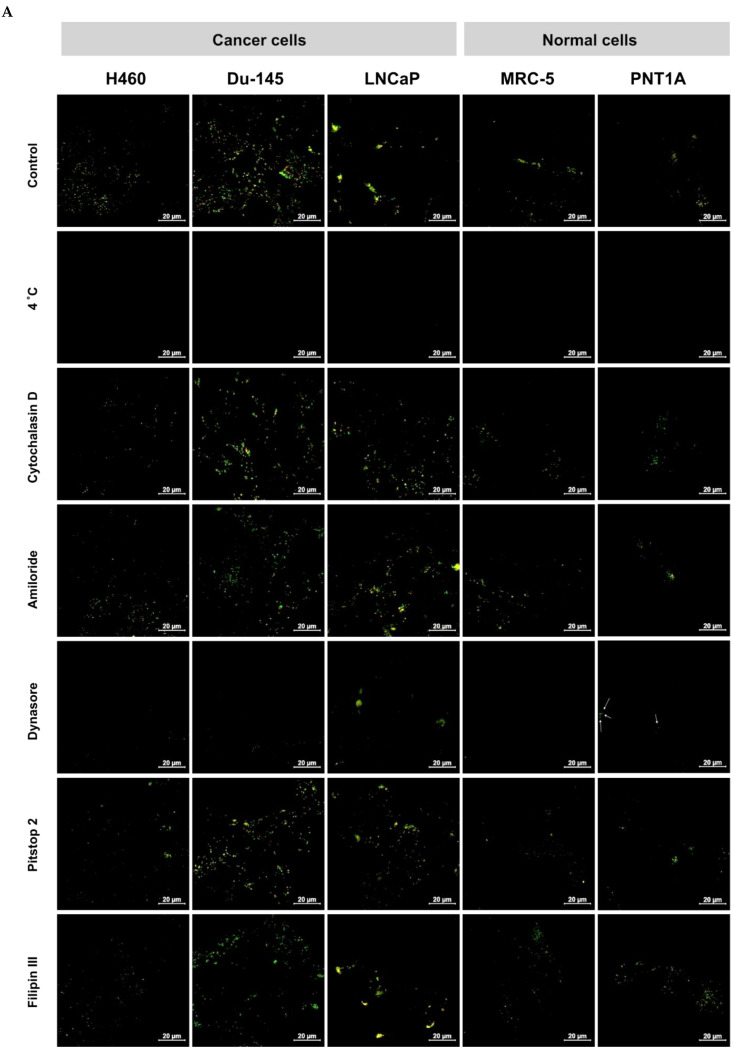

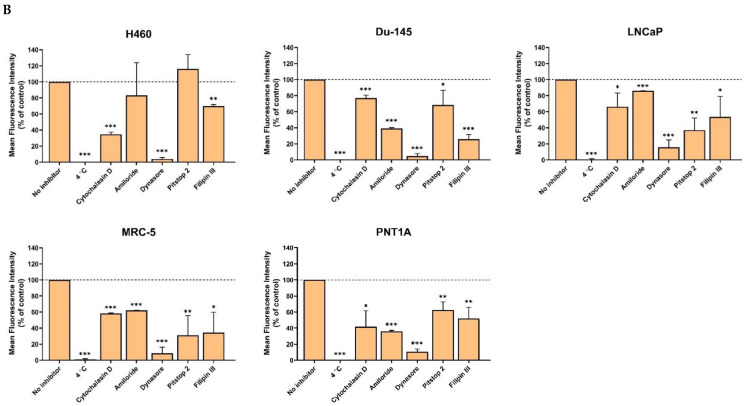
(**A**) The influence of different endocytosis inhibitors on the internalization of QD_green_-*β*-CD(C-2028)-FA in cancer and normal cells. H460, Du-145, LNCaP, MRC-5, and PNT1A cells were preincubated with drug-free medium (no inhibitor), at 4 °C, Cytochalasin D, Amiloride, Dynasore, Pitstop 2, and Filipin III for 30 min, which was followed by further incubation with QD_green_-CD(C-2028)-FA for 4 h. The internalization of QD_green_-*β*-CD(C-2028)-FA in cells was explored by CLSM. The scale bar is 20 μm. (**B**) MFI values of the panel (a) were determined using ImageJ software and normalized to control (cells treated QD_green_-*β*-CD(C-2028)-FA without inhibitor). Data are expressed as the mean ± SD, *n* = 3. * *p* < 0.05, ** *p* < 0.01, *** *p* < 0.001—statistically significant differences between the MFI of QD_green_-*β*-CD(C-2028)-FA in the cells incubated with inhibitors and control cells (no inhibitor) (Student’s *t*-test).

The CLSM studies, shown in Figure 6, indicated that the internalization of QD_green_-*β*-CD(C-2028)-FA nanoconjugate to cells of all lines was highly energy-dependent and was taken up by three endocytosis pathways: CME, CavME, and MP. However, the level of these endocytosis pathways was different depending on cell lines. The internalization of QD_green_-*β*-CD(C-2028)-FA into H460 cells treated with Amiloride and Pitstop 2 was similar to the control (without inhibitor). In contrast, preincubation with the other used inhibitors significantly decreased the internalization of this nanoconjugate to the cells. The residue of the studied cell lines (Du-145, LNCaP, MRC-5, and PNT1A) incubated with all inhibitors significantly decreased the observed cellular uptake of QD_green_-*β*-CD(C-2028)-FA nanoconjugate. Interestingly, the presence of folic acid in nanoconjugates should determine mostly the receptor-mediated endocytosis [45]. In our previous studies [27], we demonstrated that the mechanism of internalization of QD_green_-C-2028, without navigating molecules, was different, depending on the cell line. In the case of lung cancer, H460, and normal MRC-5 cells, QD_green_-C-2028 were taken up by CME, CavME, and MP. In turn, in the case of colon cancer HCT116 cells, the conjugate was taken up only by CME. Therefore, multiple endocytosis pathways QD_green_-*β*-CD(C-2028)-FA nanoconjugate can be responsible for effective cellular uptake into cells.

## 3. Materials and Methods

### 3.1. Materials

First, 1-ethyl-3-(3-dimethylaminopropyl)carbodiimide hydrochloride (EDC × HCl), 3-(4,5-dimethylthiazol-2-yl)-2,5-diphenyltetrazolium bromide (MTT), *β*-cyclodextrin (*β*-CD), 4-dimethylaminopyridine (DMAP), Amiloride, Cytochalasin D, dimethyl sulfoxide (DMSO), Dynasore, folic acid (FA), Filipin III, Pitstop 2, streptomycin, and penicillin were purchased from Sigma-Aldrich, St. Louis, MO, USA. MilliQ water was used for the preparation of all aqueous solutions (Milli-Q^®^ IQ 7005 Water Purification System, Millipore, Billerica, MA, USA). All reagents and chemicals used in experiments were of analytical grade and were used as received.

### 3.2. Methods

#### 3.2.1. Applied Techniques

The physicochemical characteristic of the nanoconjugates was performed using dynamic light scattering (DLS), zeta potential (ZP), quartz crystal microbalance with dissipation (QCM-D), and UV-vis spectroscopy. The DLS and ZP studies were carried out in 0.02 M PBS buffer pH 7.4 at 21 °C with a Zetasizer nano series apparatus (Malvern Instruments, England) with a He-Ne (4 mW) laser at 632.8 nm. The UV-vis spectra were recorded using a PerkinElmer spectrometer Lambda 25 (Waltham, MA, USA), in the range of 200–900 nm at room temperature in a quartz cuvette of 1 cm length of the optical window. The FTIR spectra were acquired in a transmission mode on a Perkin Elmer System 2000 spectrophotometer (Waltham, MA, USA) in the range of 400–4000 cm^−1^ with the spectral resolution of 4 cm^−1^. The pellets were prepared from a mixture of 300 mg of spectrally pure KBr with about 1 wt % of the sample. The QCM-D experiments were performed with QCM E4 apparatus (Q-sense, Biolin Scientific AB, Stockholm, Sweden) using 4.95 MHz gold quartz crystals. Before the experiments, gold crystals were cleaned in TL1 mixture (ultrapure water, 25% NH_3_(*aq*) and 30% H_2_O_2_; 5:1:1 (*v*/*v*)) at 75 °C for 5 min. Then, the crystals were rinsed thoroughly with ethanol and distilled water and dried with a stream of argon.

#### 3.2.2. Synthesis of *β*-Cyclodextrin Functionalized Quantum Dots

The procedure of the synthesis of quantum dots (QD_green_, Ag_1.0_In_1.2_Zn_5.6_S_9.4_; QD_red_, Ag_1.0_In_1.0_Zn_1.0_S_3.5_) is described in detail in our previous paper [25]. The dispersion of quantum dots (*ca.* 16 mg × mL^−1^; 3 mL) was diluted with distilled water (10 mL), and then, the 1-ethyl-3-(3-dimethylaminopropyl)carbodiimide hydrochloride (EDC × HCl; 76.8 mg, 0.4 mmol) was added, and the reaction mixture was stirred at room temperature for 15 min. A DMSO (2 mL) solution containing *β*-cyclodextrin (226.8 mg, 2.0 mmol) and 4-dimethylaminopyridine (DMAP; 12.2 mg, 0.1 mmol) was slowly added, and the reaction mixture was stirred at room temperature for 48 h. Then, it was dialyzed (MWCO 2 kDa) against distilled water for 7 days to provide target *β*-cyclodextrin functionalized quantum dots (QDs-*β*-CD).

#### 3.2.3. Synthesis of Folic Acid Functionalized *β*-Cyclodextrin Containing Quantum Dots

**Figure 7:**
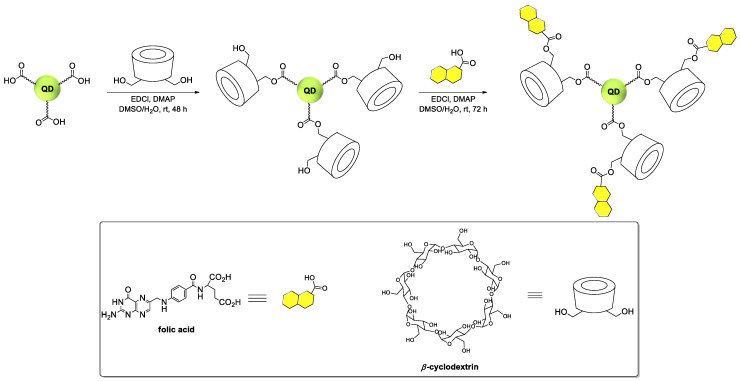
Synthesis of folic acid functionalized *β*-cyclodextrin containing quantum dots.

A DMSO (7 mL) solution containing folic acid (88.2 mg, 0.20 mmol) and EDC × HCl (58.0 mg, 0.30 mmol) was stirred at room temperature for 30 min. Then, it was added dropwise to a stirred aqueous dispersion of QDs-*β*-CD and DMAP (18.3 mg, 0.15 mmol). The reaction mixture was stirred at room temperature for 72 h. Then, it was dialyzed (MWCO 2 kDa) against distilled water for 14 days to provide target folic acid (FA) functionalized *β*-cyclodextrin containing quantum dots (QDs-*β*-CD-FA). The scheme of the procedure synthesis of the QDs-*β*-CD-FA nanoconjugates is shown in Figure 7.

**Figure 8:**
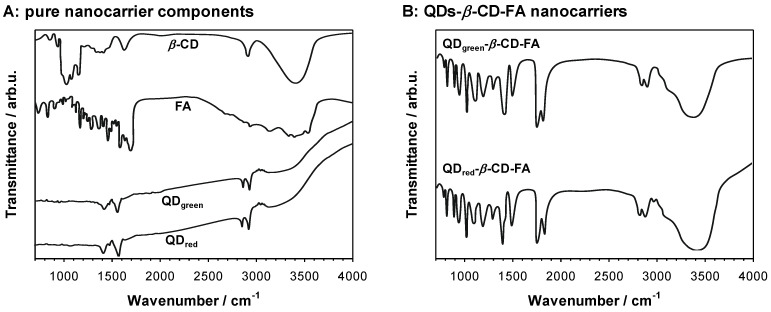
FTIR spectra obtained for pure nanocarrier components (**A**) and synthesized QDs-*β*-CD-FA nanocarriers (**B**).

The principle of the chemical reactions in the synthesis of QDs-*β*-CD-FA nanoconjugates was the formation of an ester bond. The successful attachment of *β*-CD to QDs and folic acid to *β*-CD was confirmed by FTIR analysis. The FTIR spectra for pure nanoconjugates components and nanoconjugates are shown in Figure 8. The strongest bands that appear at *ca*. 1730 cm^−1^ (Figure 8B) can be assigned -C=O- stretching vibrations in the ester group. The presence of other characteristic bands of all components in the nanoconjugates spectra confirmed the correctness of the applied synthesis. The UV-vis studies of QDs-*β*-CD-FA nanoconjugates allowed us to determine the amount of introduced FA to the nanoconjugate. The measurements were performed for 50 µM FA solution and 1.0 mg × mL^−1^ QDs-*β*-CD-FA. By comparison the absorbance value at *ca*. 359 nm, according to the Lambert–Beer equation, the concentration of FA in nanoconjugate was calculated and was equaled 25.3 µM ≡ 11.2 mg × mL^−1^.

#### 3.2.4. Selective Loading of C-2028 into QD-*β*-CD-FA Nanoconjugates

**Figure 9:**
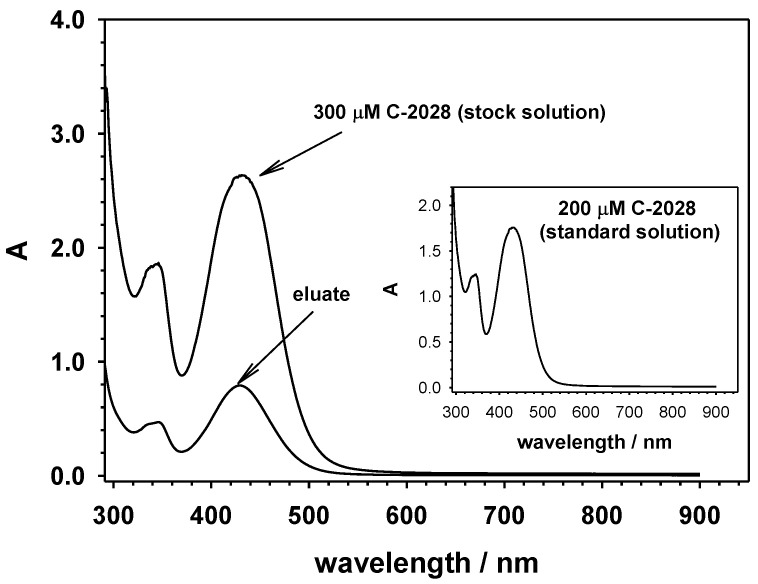
UV-vis spectra of C-2028 before (stock solution) and after interaction with QDs-*β*-CD-FA nanoconjugates (eluate). Inset: UV-vis spectrum of C-2028 standard solution (200 μM).

The size-selective method of loading C-2028 into QDs-*β*-CD-FA nanoconjugates was based on the inclusion phenomenon. The size of the C-2028 allows it to be closed encapsulated inside the *β*-CD cavity (pocket size 7.9 × 7.0 Å) [46]. The 10 mL of water mixture containing QDs-*β*-CD-FA nanoconjugates (1.0 mg·mL^−1^) and C-2028 (300 µM) was incubated overnight with stirring in ThermoMixer at room temperature. Then, the mixture was dialyzed (5 times; Float-A-Lyzer^®^ G2 Dialysis Device 0.5–1 K MWCO) against distilled water to remove unbound C-2028 molecules. To determine the amount of C-2028 loaded into *β*-CD, the eluate of the dialysis steps was collected and analyzed by UV-vis spectroscopy; see Figure 9. To be sure that the Lambert–Beer law can be applied in the determination of C-2028 concentration in the nanoconjugate, the UV-vis spectra of pure C-2028 compound was recorded in the concentration range 1–400 μM. The obtained dependence *A* = f(*C*) was linear in the whole examined concentration range (*A* = (0.0087 ± 0.0000092)*C* + (0.0026 ± 0.0012); *r*^2^ = 0.9999). The linear relationship *A* versus *C* confirmed that in the studied concentration range, no side reactions between C-2028 molecules take place. The amount of the C-2028 compound complexed with cyclodextrin was calculated from the difference in the intensity of the absorption at 430 nm for the stock solution and the eluate. This difference was comparable with the absorption intensity obtained for 200 μM C-2028 standard solution (inset in Figure 9). It was found that 70% (*ca.* 210 µM) of C-2028 formed an inclusion complex with *β*-CD, whereas 30% was washed off.

#### 3.2.5. Cell Culture

Human cancer (H460, Du-145, and LNCaP) and normal (MRC-5) cell lines were purchased from the American Type Culture Collection (ATCC; Manassas, VA, USA). The PNT1A normal cell line was provided by Prof. Jędrzej Antosiewicz from the Medical University of Gdańsk (Gdańsk, Poland). Cell lines were tested negatively for mycoplasma using a Universal Mycoplasma Detection Kit—ATCC-30-1012 K (ATCC). H460, LNCaP, and PNT1A cells were cultured in RPMI 1640 medium (Sigma-Aldrich, St. Louis, MO, USA). Du-145 and MRC-5 cells were cultured in EMEM medium (Eagle’s Minimal Essential Medium, Sigma-Aldrich, St. Louis, MO, USA). Both media contained 10% fetal bovine serum (FBS; Biowest, Riverside, MO, USA), 100 µg × mL^−1^ of streptomycin, and 100 unit × mL^−1^ of penicillin. In the case of MRC-5 cells, the medium was supplemented only with 10% FBS, without antibiotics. All cells were incubated in a humidified atmosphere containing 5% CO_2_ at 37 °C. Experiments were performed with cells in the exponential phase of growth.

#### 3.2.6. MTT Assay

The in vitro cytotoxicity of QD_green/red,_ *β*-CD, QD_green/red_-*β*-CD-FA, C-2028, *β*-CD(C-2028), QD_green/red_-C-2028, and QD_green/red_-*β*-CD-FA-C-2028 nanoconjugates was assessed by MTT assay. Briefly, cells (2 × 10^4^ cells/well) for H460 and Du-145 (3 × 10^4^ cells/well) for LNCaP and PNT1A (4.5 × 10^4^ cells/well) for MRC-5 were seeded on 24-well plates. After 24 h of preincubation at 37 °C in a humidified 5% CO_2_ atmosphere, the culture medium was removed and replaced by a fresh medium containing different concentrations of QD_green/red_, *β*-CD, QD_green/red_-*β*-CD-FA, C-2028, *β*-CD(C-2028), QD_green/red_-C-2028, and QD_green/red_-*β*-CD(C-2028)-FA nanoconjugates. Following 72 h of incubation, 200 μL of MTT solution (4 mg × mL^−1^) was added to each well containing 2 mL of media and incubated at 37 °C for 3 h. Furthermore, media containing MTT solution were removed, and the formazan crystals were dissolved in 1 mL of DMSO shaking the plates for 30 min. Finally, the absorbance at 540 nm was detected using a microplate reader (iMark™, Bio-Rad, USA). Cytotoxicity was expressed as IC_50_ and IC_80_ values. A dose–response curve was plotted and used to calculate drug concentration that yielded 50 and 80% inhibition of cell growth (IC_50_ and IC_80_), respectively. Results were obtained using three independent experiments (*n* = 3).

#### 3.2.7. Confocal Microscopy Imaging

Fluorescence properties of QD_green_ and C-2028 were used. They served as an intrinsic fluorescence probe to efficiently explore their cellular uptake. Briefly, 1 × 10^6^ cells were seeded in the 60 mm plate with glass coverslips (except control, QD_green_, *β*-CD, and QD_green_-*β*-CD-FA for 48 and 72 h—5 × 10^5^ and 2 × 10^5^ cells in the case of cancer cells and 8 × 10^5^ and 6 × 10^5^ cells in the case of normal cells were seeded, respectively) and incubated overnight. Next, cells were treated with QD_green_, *β*-CD, QD_green_-*β*-CD-FA, C-2028, *β*-CD(C-2028), QD_green_-C-2028, and QD_green_-*β*-CD(C-2028)-FA nanoconjugates at IC_80_ values corresponding to C-2028 alone for 1, 24, 48, and 72 h of incubation (cellular uptake studies). The concentration (IC_80_ value) used in the experiments for C-2028, *β*-CD(C-2028), QD_green_-C-2028, and QD_green_-*β*-CD(C-2028)-FA nanoconjugates were 0.035 μM for H460 and MRC-5, 0.024 μM for Du-145, and 0.133 μM for LNCaP and PNT1A cell lines, respectively. The concentration of QD_green_, *β*-CD, and QD_green_-CD-FA, corresponding to the IC_80_ value of C-2028 alone in the nanoconjugates was 0.0009 mg × mL^−1^ for H460 and MRC-5, 0.0008 mg × mL^−1^ for Du-145, and 0.0044 mg × mL^−1^ for LNCaP and PNT1A cell lines, respectively. Furthermore, to explore the mechanism of internalization of QD_green_-*β*-CD(C-2028)-FA nanoconjugate, cells were first preincubated with different inhibitors (at concentrations that were non-toxic to the cells): drug-free medium (no inhibitor), at 4 °C, 5 µM Cytochalasin D, 30 µM Amiloride, 80 µM Dynasore, 25 µM Pitstop 2 and 1.5 µM Filipin III for 30 min, followed by further incubation with QD_green_-*β*-CD(C-2028)-FA nanoconjugate for 4 h. Next, cells were washed twice in cold PBS and immediately observed using the CLSM (63× magnification; ZEISS LSM 800, Magdeburg, Germany) in all types of experiments. The imaging conditions were QD_green_ (excitation 300 nm, emission 543 nm), C-2028 (excitation 528 nm, emission 553 nm). The Mean Fluorescence Intensity (MFI) of C-2028 from the images of cells treated with C-2028 alone, *β*-CD(C-2028), QD_green_-C-2028, and QD_green_-*β*-CD(C-2028)-FA nanoconjugates, was performed with ImageJ software (version 1.8.0., Madison, WI, USA).

#### 3.2.8. Stability Analysis

Stability studies of QDs-*β*-CD(C-2028)-FA nanoconjugates were performed with DLS and OCM-D measurements at room temperature for 7 days, every 24 h. Before placing the samples in the DLS measuring chamber, the solutions were gently mixed. Between the measurements, the samples were stored in the refrigerator (4 °C). The DLS studies allowed us to control the size of the nanoconjugates as a function of time and type of environment. The size was controlled in water, PBS buffer, and EMEM medium. To get information about the possible release of C-2028 from the nanoconjugate, the gravimetry was applied. In this study, the nanoconjugates (QDs-*β*-CD(C-2028)-FA) were physically immobilized on the quartz crystal surface. The 100 μL droplet of QDs-*β*-CD(C-2028)-FA nanoconjugate in the concentration of 5.0 × 10^−3^ mg × mL^−1^ was placed on the sensor and left to dry in the desiccator. After the droplets were dry, the crystal was placed in the QCM-D E4 chamber. The measurements were performed in the flow system (flow rate 100 μL × min^−1^) for different media (water, PBS buffer, and EMEM medium).

#### 3.2.9. Statistical Analysis

Data were expressed as a mean with standard deviation (SD) and collected from at least three independent experiments. Statistical analysis was performed by the Student’s *t*-test, and the differences of *p* < 0.05 between the two groups were considered as statistically significant: * *p* < 0.05, ** *p* < 0.01, *** *p* < 0.001.

## 4. Conclusions

In this study, we presented a new, efficient transporting platform for an anticancer drug—nanoconjugate of quantum dots and *β*-cyclodextrin labeled with a self-navigating molecule: folic acid (QDs-*β*-CD-FA). The conjugation of unsymmetrical bisacridines derivative C-2028 (potential anticancer drug) with non-toxic QDs or QDs-*β*-CD-FA did not change the cytotoxic activity of this compound against cancer and normal cells. In studied cell lines, we did not observe the beneficial influence of the self-navigating molecule (FA) on cytotoxicity, which is desirable in cancer therapy. In the future, there is a need to check different cell lines with the overexpression of FRs to confirm this thesis. However, confocal microscopy images showed that the use of FA in conjugates significantly increased the amount of delivered compound to the cells, especially in the case of cancer cells. The amount of bound C-2028 to 1 g of nanoconjugate was 6.16 and 4.74 mg for QD_green_-*β*-CD-FA and QD_red_-*β*-CD-FA, respectively. CLSM analysis showed that the presence of folic acid in nanoconjugates significantly increased the amount of C-2028 in the cells, compared to C-2028 alone and QD_green_-C-2028, especially in the case of cancer Du-145 and H460 cells. The effective cellular uptake of the QD_green_-*β*-CD(C-2028)-FA nanoconjugate may be associated with multiple pathways of endocytosis, including MP, CME, and CavME. The efficiency of internalization QD_green_-*β*-CD(C-2028)-FA nanoconjugate through these pathways was different, depending on cell lines. In summary, the described results demonstrated that the use of FA may serve as a good self-navigating molecule in the QDs platform for drug delivery to cancer cells.

## Figures and Tables

**Table 1:** Values of hydrodynamic diameter and PDI of QDs-*β*-CD(C-2028)-FA nanoconjugates (5.0 × 10^−3^ mg × mL^−1^) in various media in the function of time (1–7 days).

Type of Measurement	Water		PBS		EMEM Medium	
	QD_green_-*β*-CD (C-2028)-FA	QD_red_-*β*-CD (C-2028)-FA	QD_green_-*β*-CD (C-2028)-FA	QD_red_-*β*-CD (C-2028)-FA	QD_green_-*β*-CD (C-2028)-FA	QD_red_-*β*-CD (C-2028)-FA
**size**	1st: 145 ± 19	1st: 149 ± 10	1st: 156 ± 23	1st: 167 ± 27	1st: 189 ± 31 (96%) 856 ± 45 (4%)	1st: 191 ± 36 (95%) 925 ± 41 (5%)
	2nd: 148 ± 15	2nd: 147 ± 12	2nd: 160 ± 25	2nd: 171 ± 30	2nd: 195 ± 29 (97%) 840 ± 52 (3%)	2nd: 199 ± 38 (92%) 964 ± 33 (8%)
	3rd: 151 ± 23	3rd: 149 ± 11	3rd: 162 ± 27	3rd: 175 ± 29	3rd: 197 ± 36 (92%) 920 ± 24 (8%)	3rd: 205 ± 33 (89%) 1003 ± 45 (11%)
	4th: 146 ± 21	4th: 153 ± 14	4th: 161 ± 24	4th: 180 ± 33	4th: 192 ± 24 (93%) 950 ± 65 (7%)	4th: 204 ± 41 (87%) 1019 ± 67 (13%)
	5th: 154 ± 18	5th: 152 ± 16	5th: 164 ± 25	5th: 186 ± 27	5th: 199 ± 28 (89%) 894 ± 71 (11%)	5th: 210 ± 37 (81%) 1035 ± 73 (19%)
	6th: 156 ± 17	6th: 154 ± 14	6th: 167 ± 29	6th: 192 ± 31	6th: 200 ± 32 (85%) 980 ± 29 (15%)	6th: 212 ± 44 (79%) 1106 ± 62 (21%)
	7th: 159 ± 26	7th: 155 ± 15	7th: 170 ± 31	7th: 195 ± 35	7th: 206 ± 33 (83%) 905 ± 47 (17%)	7th: 215 ± 42 (90%) 1134 ± 58 (20%)
**PDI**	1st: 0.105	1st: 0.118	1st: 0.120	1st: 0.125	1st: 0.180	1st: 0.201
	2nd: 0.108	2nd: 0.121	2nd: 0.119	2nd: 0.129	2nd: 0.192	2nd: 0.250
	3rd: 0.111	3rd: 0.120	3rd: 0.123	3rd: 0.135	3rd: 0.205	3rd: 0.307
	4th: 0.104	4th: 0.119	4th: 0.127	4th: 0.133	4th: 0.262	4th: 0.355
	5th: 0.106	5th: 0.123	5th: 0.124	5th: 0.135	5th: 0.297	5th: 0.390
	6th: 0.109	6th: 0.125	6th: 0.129	6th: 0.139	6th: 0.315	6th: 0.431
	7th: 0.112	7th: 0.127	7th: 0.131	7th: 0.138	7th: 0.352	7th: 0.468

**Table 2:** IC_50_ and IC_80_ values for C-2028 alone, and its nanoconjugates: *β*-CD(C-2028), QD_green_-C-2028, QD_green_-*β*-CD(C-2028)-FA, QD_red_-C-2028, as well as QD_red_-*β*-CD(C-2028)-FA determined in human H460, Du-145, LNCaP, MRC-5, and PNT1A cell lines. * *p* < 0.05, ** *p* < 0.001, *** *p* < 0.0001, statistically significant differences between the cytotoxicity of nanoconjugates and the compound (C-2028) alone (Student’s *t*−test), *n* = 3; *n.d.* (not determined).

Compound		Cell Line				
		H460	Du-145	LNCaP	MRC-5	PNT1A
**C-2028**	**IC_50_**	0.016 ± 0.001	0.009 ± 0.001	0.066 ± 0.004	0.018 ± 0.002	0.028 ± 0.012
	**IC_80_**	0.035 ± 0.003	0.024 ± 0.002	0.133 ± 0.004	0.138 ± 0.006	1.14 ± 0.32
***β*-CD(C-2028)**	**IC_50_**	0.054 ± 0.007 **	0.033 ± 0.017 *	0.132 ± 0.039 *	0.109 ± 0.044	0.033 ± 0.010
	**IC_80_**	0.120 ± 0.003 **	0.370 ± 0.149 *	0.654 ± 0.055 ***	*n.d.*	2.52 ± 0.59 *
**QD_green_-C-2028**	**IC_50_**	0.018 ± 0.005	0.010 ± 0.001	0.088 ± 0.015	0.082 ± 0.006 **	0.104 ± 0.022 **
	**IC_80_**	0.083 ± 0.024 *	0.028 ± 0.002 *	0.155 ± 0.027	0.184 ± 0.060	*n.d.*
**QD_green_-*β*-CD(C-2028)-FA**	**IC_50_**	0.020 ± 0.003	0.008 ± 0.001	0.044 ± 0.003 **	0.057 ± 0.011	0.064 ± 0.014 *
	**IC_80_**	0.060 ± 0.004	0.022 ± 0.004	0.096 ± 0.002 ***	0.156 ± 0.022	0.265 ± 0.044 *
**QD_red_-C-2028**	**IC_50_**	0.037 ± 0.008 *	0.019 ± 0.002 ***	0.072 ± 0.013	0.081 ± 0.003 ***	0.021 ± 0.006
	**IC_80_**	0.097 ± 0.008 **	0.101 ± 0.017 ***	0.160 ± 0.044	0.250 ± 0.067 *	0.835 ± 0.131
**QD_red_-*β*-CD(C-2028)-FA**	**IC_50_**	0.010 ± 0.003	0.012 ± 0.006	0.048 ± 0.008	0.041 ± 0.009 *	0.060 ± 0.007 *
	**IC_80_**	0.040 ± 0.013	0.084 ± 0.008 ***	0.092 ± 0.008 **	0.091 ± 0.005 ***	0.258 ± 0.016 *

## Data Availability

Data are contained within the article, Supplementary Material, and Most Wiedzy Portal. The DOI number of raw data in Most Wiedzy Portal are: 10.34808/n1mr-e516; 10.34808/p6t9-rx05; 10.34808/g23f-8x42; 10.34808/1ej4-zr38; 10.34808/s722-j991; 10.34808/j4zk-4h41; 10.34808/3xfj-8j61; 10.34808/tght-ye55; 10.34808/tkbt-6568; 10.34808/zsy0-az13.

## Supplementary Materials

The following supporting information can be downloaded at: https://www.mdpi.com/article/10.3390/ijms23031261/s1.
