# Supplementary material for: Foliate-Targeting Quantum Dots-β-Cyclodextrin Nanocarrier for Efficient Delivery of Unsymmetrical Bisacridines to Lung and Prostate Cancer Cells
Source: Int J Mol Sci. 2022 Jan 23;23(3):1261. doi: 10.3390/ijms23031261 (PMC8835877; doi:10.3390/ijms23031261)
Supplement: Supplementary file 1 [file ijms-23-01261-s001.zip › ijms-1537067-supplementary.pdf]

International Journal of *Molecular Sciences*

MDPI

# Supplementary Materials

## Foliate-targeting quantum dots–*β*–cyclodextrin nanocarrier for efficient delivery of unsymmetrical bisacridines to lung and prostate cancer cells

**Joanna Pilch [1,*], Patrycja Kowalik [2,3], Agata Kowalczyk [2], Piotr Bujak [3], Artur Kasprzak [3], Ewa Paluszkiewicz [1], Ewa Augustin [1], and Anna M. Nowicka [2,*]**

[1] Faculty of Chemistry, Gdańsk University of Technology, Narutowicza Str. 11/12, 80-233 Gdańsk, Poland; joapilch@pg.edu.pl (J.P.); ewa.paluszkiewicz@pg.edu.pl (E.P.); ewa.augustin@pg.edu.pl (E.A.);

[2] Faculty of Chemistry, University of Warsaw, Pasteura Str. 1, 02-093 Warsaw, Poland; patrycja.kowalik@student.uw.edu.pl (P.K.); akowalczyk@chem.uw.edu.pl (A.K.); anowicka@chem.uw.edu.pl (A.M.N.);

[3] Faculty of Chemistry, Warsaw University of Technology, Noakowskiego Str. 3, 00-664 Warsaw, Poland; pkowalik@ch.pw.edu.pl (P.K.); piotrbujakchem@poczta.onet.pl (P.B.), akasprzak@ch.pw.edu.pl (Ar.K.)

* Correspondence: joapilch@pg.edu.pl (J.P.); anowicka@chem.uw.edu.pl (A.M.N.)

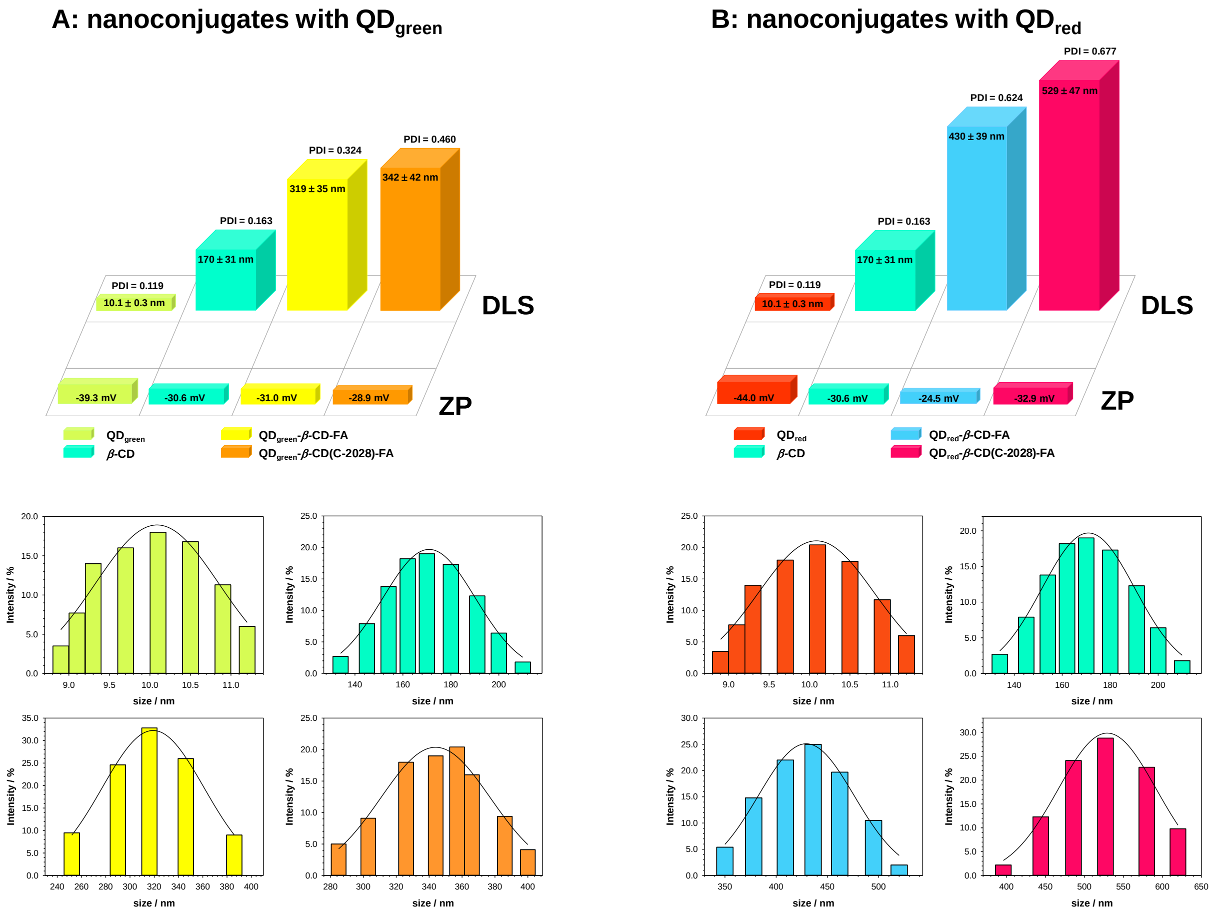


**Figure S1.** Hydrodynamic diameter, zeta potential, and normal distribution diagram of size for QDs, $\beta$–CD, QDs–$\beta$–CD–FA, and QDs–$\beta$–CD(C–2028)–FA nanoconjugates obtained in 0.02 M PBS buffer. Experimental conditions:; $C_{QD\text{-}\beta\text{-}CD\text{-}FA}$ = 1.0 mg·mL$^{-1}$; $C_{C\text{-}2028}$ = 210 µM; $C_{\beta\text{-}CD}$ = 1.0 mg·mL$^{-1}$.

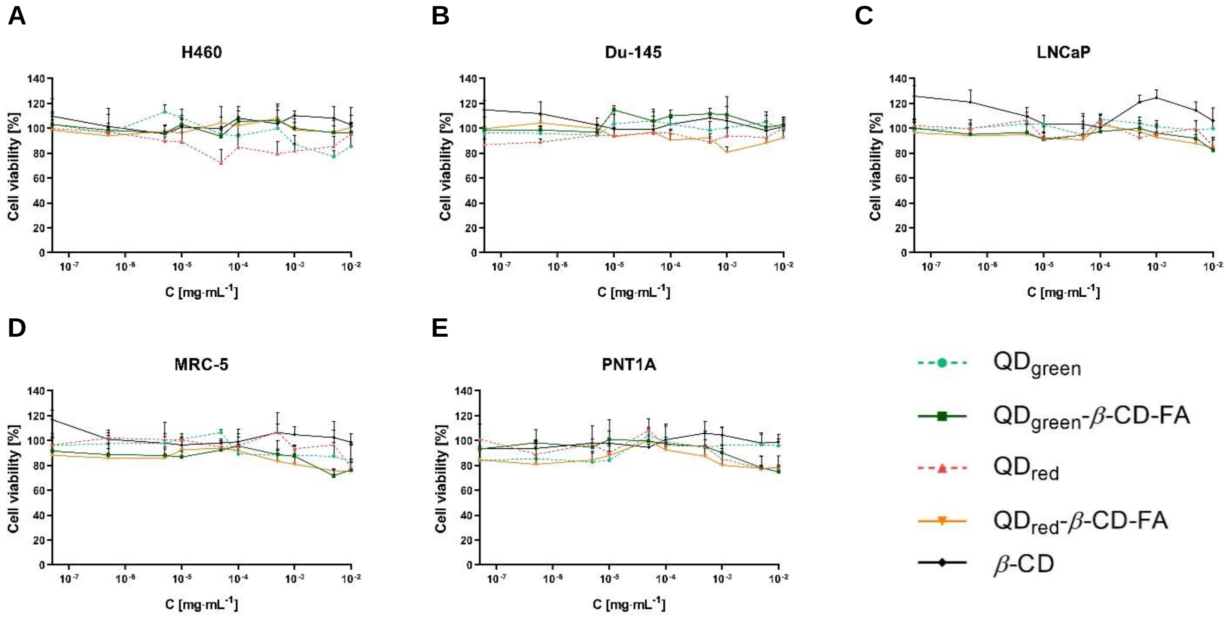


**Figure S2.** Growth inhibition curves of human cancer (**A**) H460, (**B**) Du-145, and (**C**) LNCaP cells as well as normal (**D**) MRC-5 and (**E**) PNT1A cells treated with increasing concentration of β−CD, $QD_{green}$, $QD_{green}$−β−CD−FA, $QD_{red}$, and $QD_{red}$−β−CD−FA following 72 h of incubation. Data are expressed as the mean ± of three independent experiments.

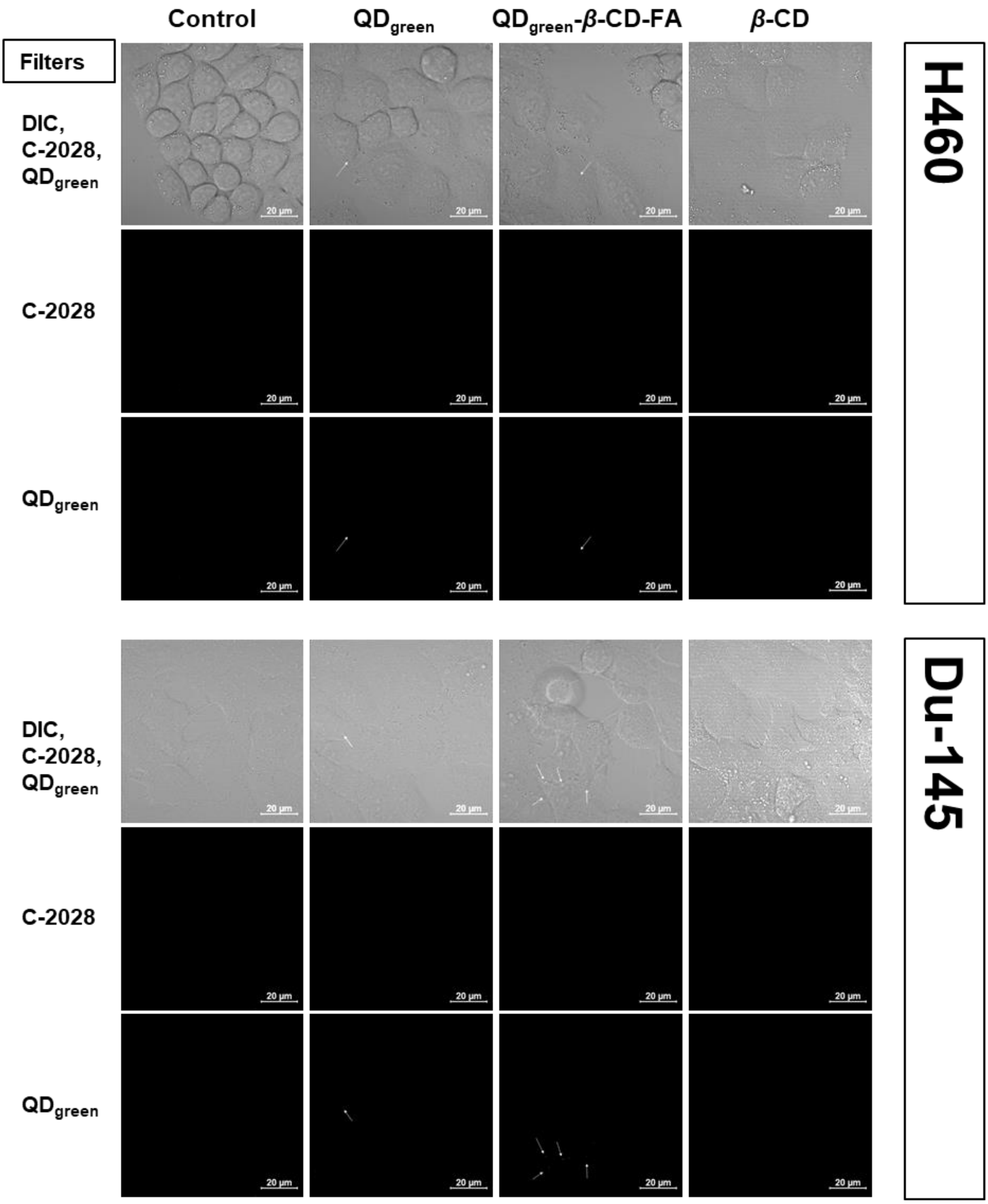
Control
QDgreen
QDgreen-β-CD-FA
β-CD
Filters
DIC,
C-2028,
QDgreen
C-2028
QDgreen
H460
Du-145
20 µm

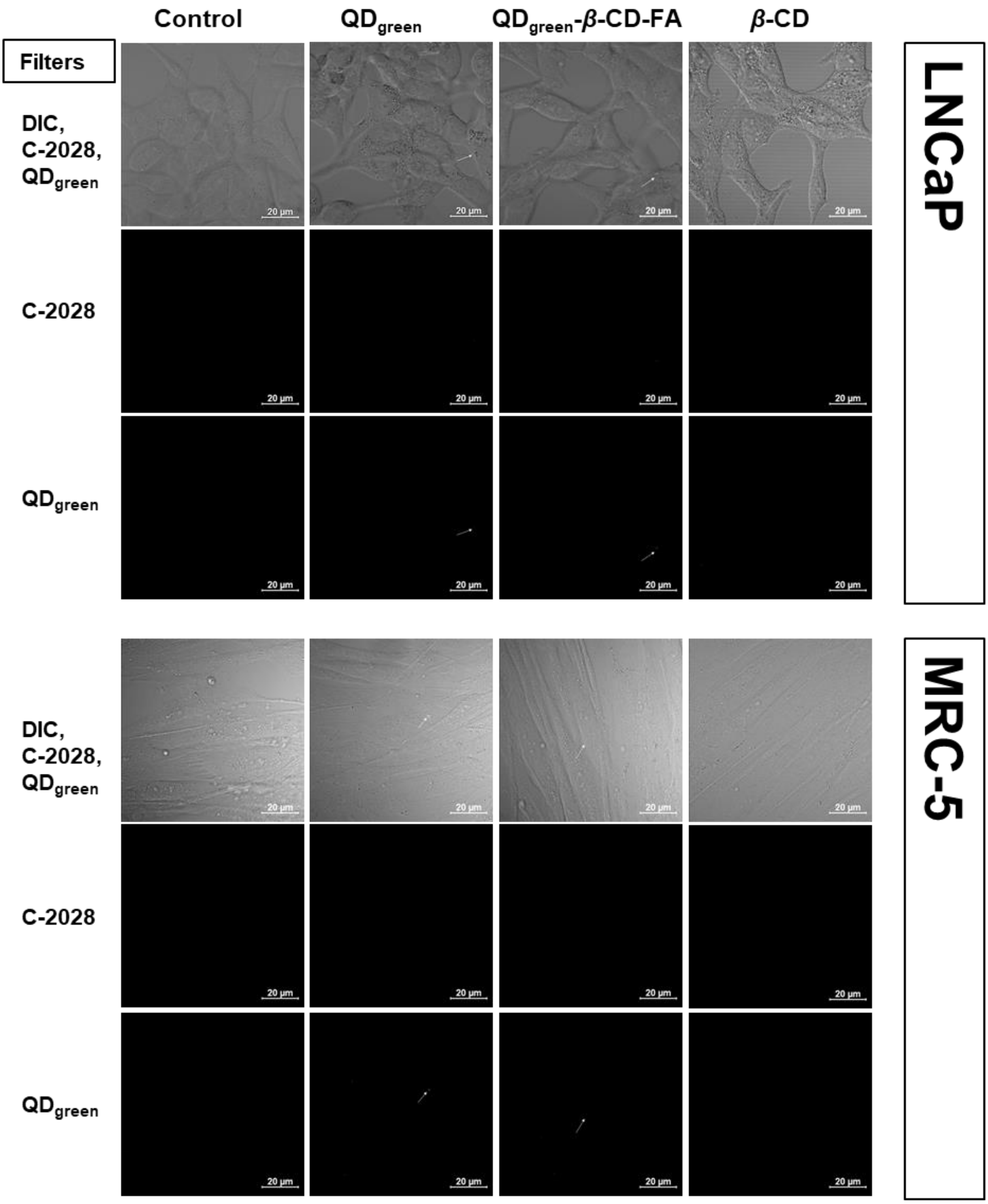
Control
QDgreen
QDgreen-β-CD-FA
β-CD
Filters
DIC, C-2028, QDgreen
C-2028
QDgreen
LNCaP
MRC-5
20 μm

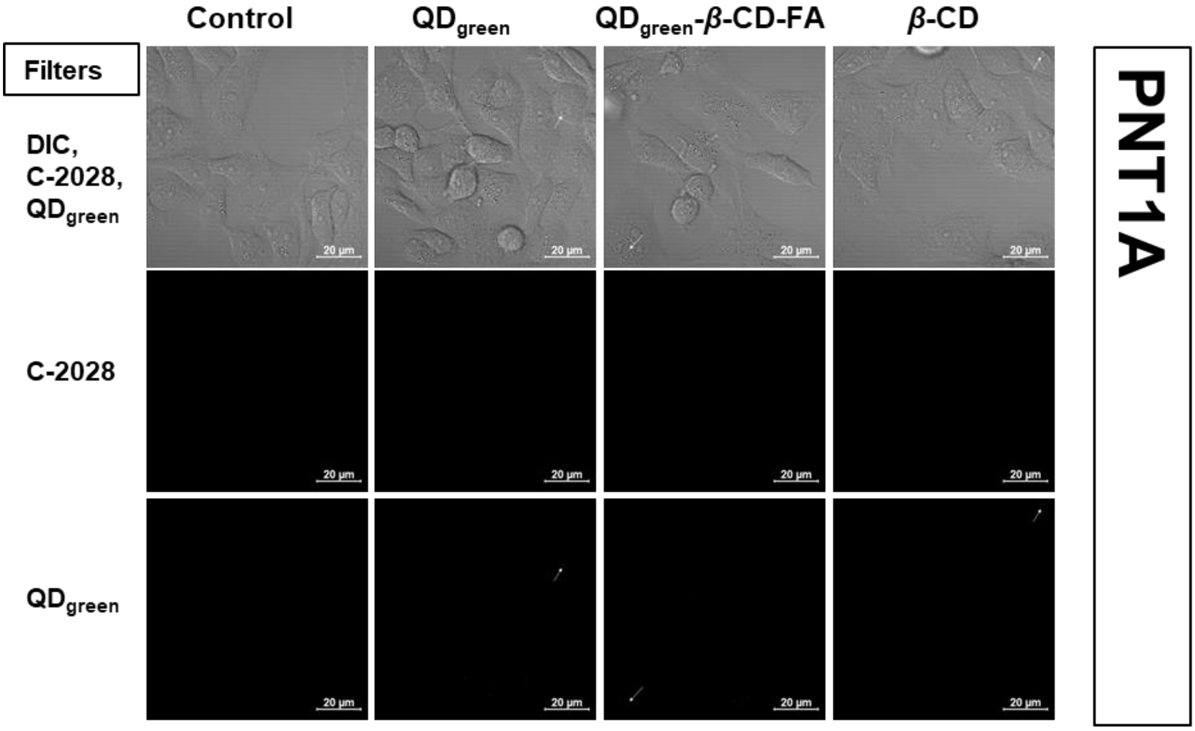


**Figure S3.** Confocal fluorescence micrographs of H460, Du-145, LNCaP, MRC-5, and PNT1A cells after 72 h of treatment with $QD_{green}$, $QD_{green}$-β–CD-FA, and β–CD. Individual signals from different filters (DIC, for C-2028, and $QD_{green}$) were indicated with a white arrow. The scale bar is 20 µm. Data are representative of three independent experiments.

A

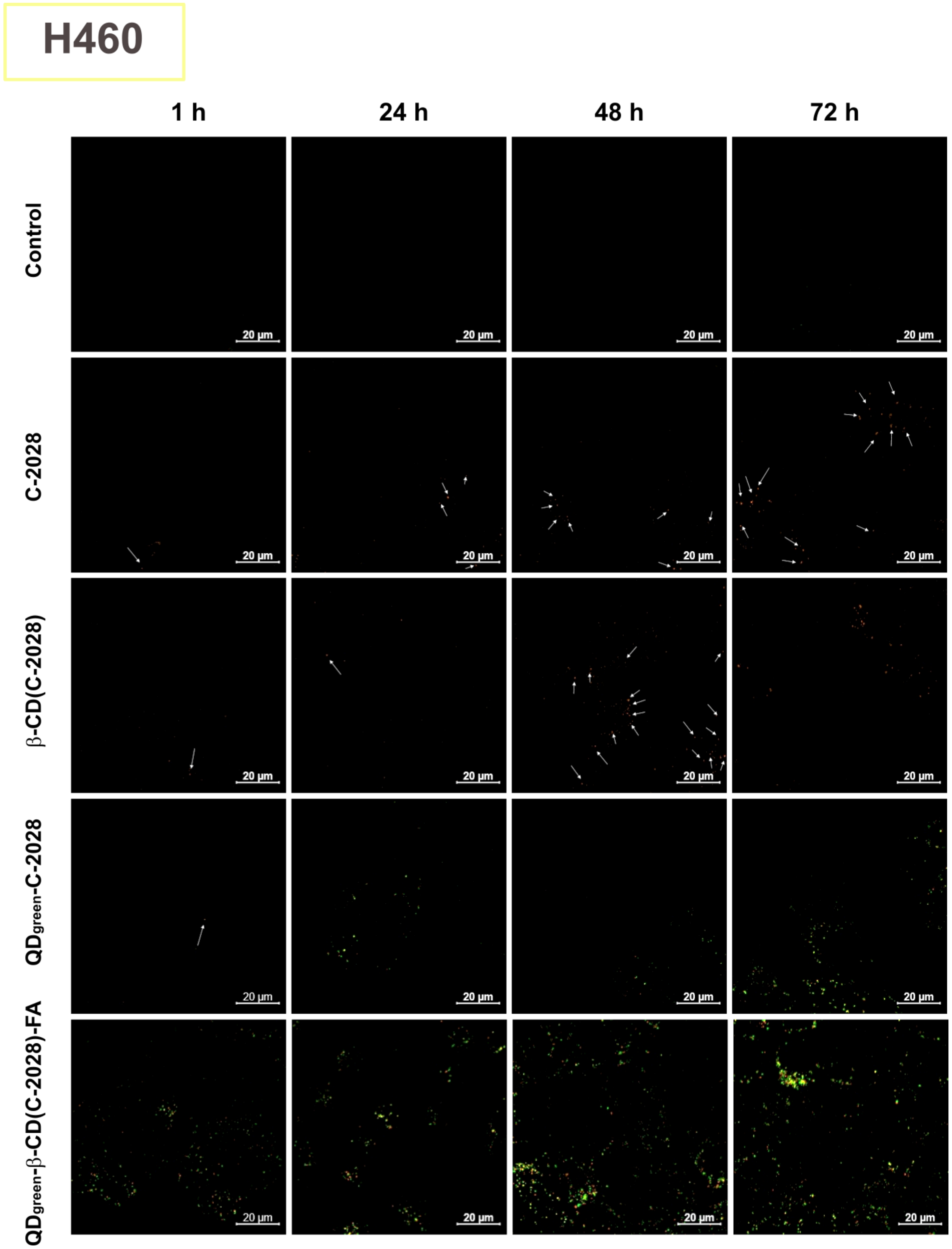
H460
1 h
24 h
48 h
72 h
Control
C-2028
β-CD(C-2028)
QDgreen-C-2028
QDgreen-β-CD(C-2028)-FA
20 µm

B

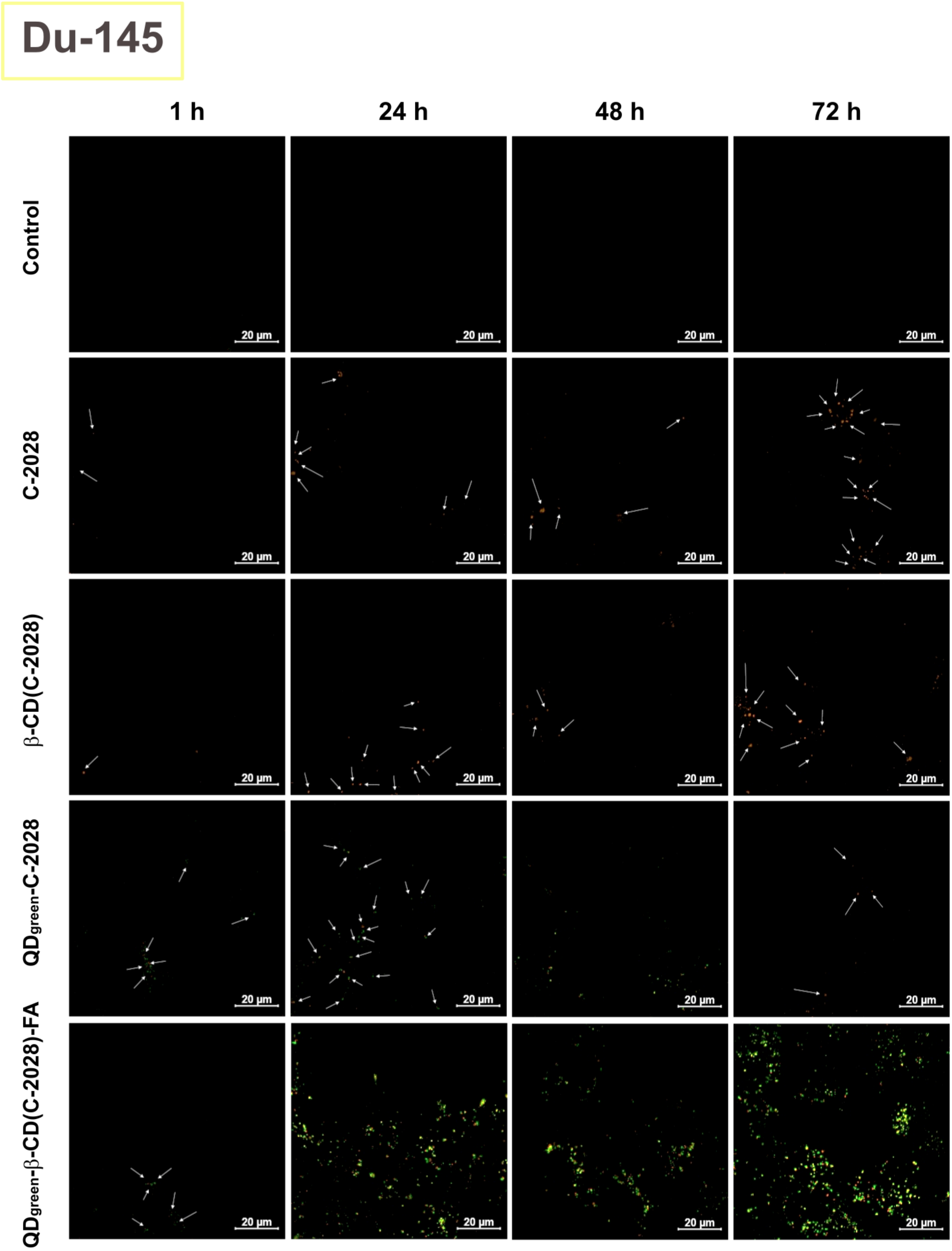

Du-145
1 h
24 h
48 h
72 h
Control
C-2028
β-CD(C-2028)
QDgreen-C-2028
QDgreen-β-CD(C-2028)-FA
20 μm

C

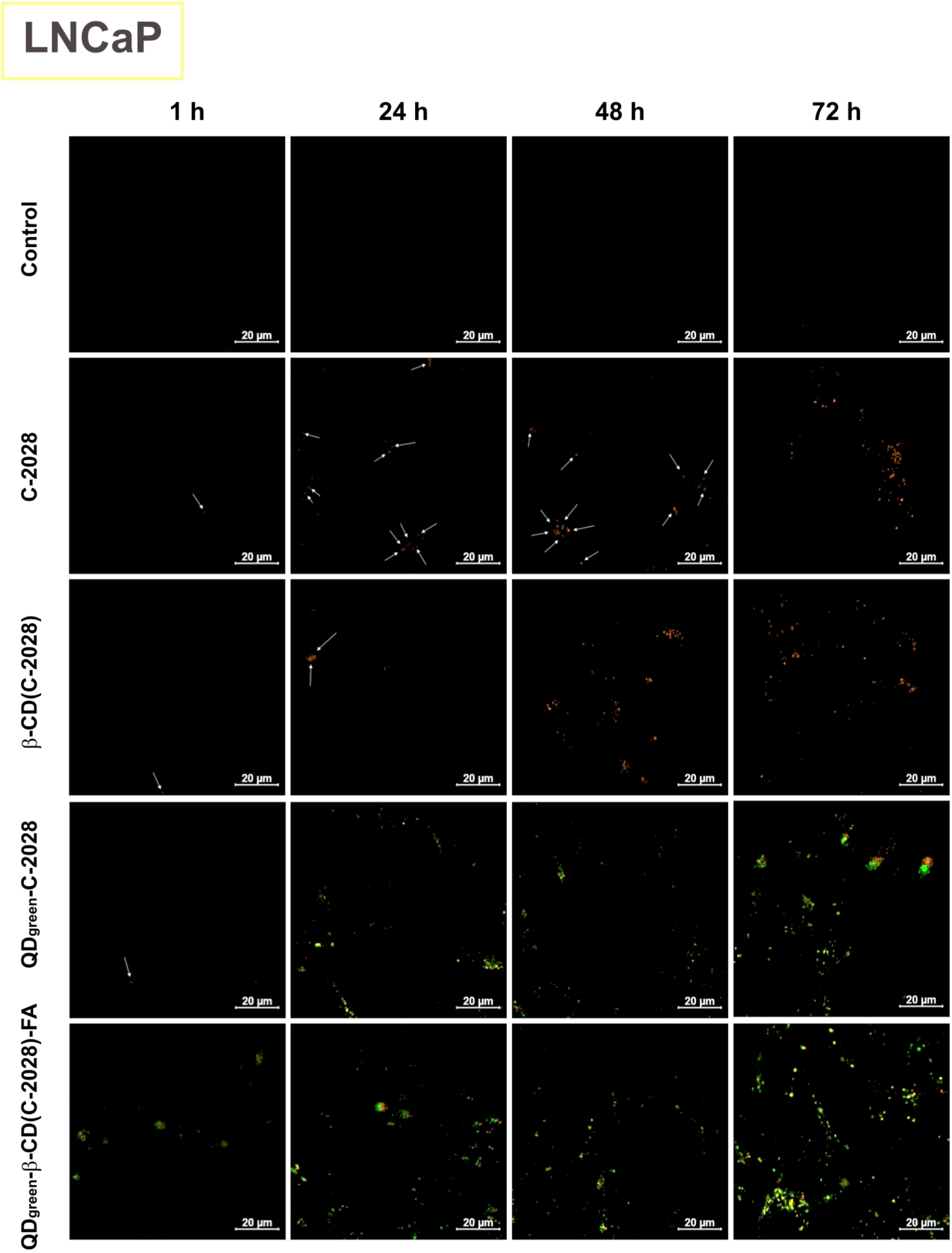

LNCaP
1 h
24 h
48 h
72 h
Control
C-2028
β-CD(C-2028)
QDgreen-C-2028
QDgreen-β-CD(C-2028)-FA
20 μm

D

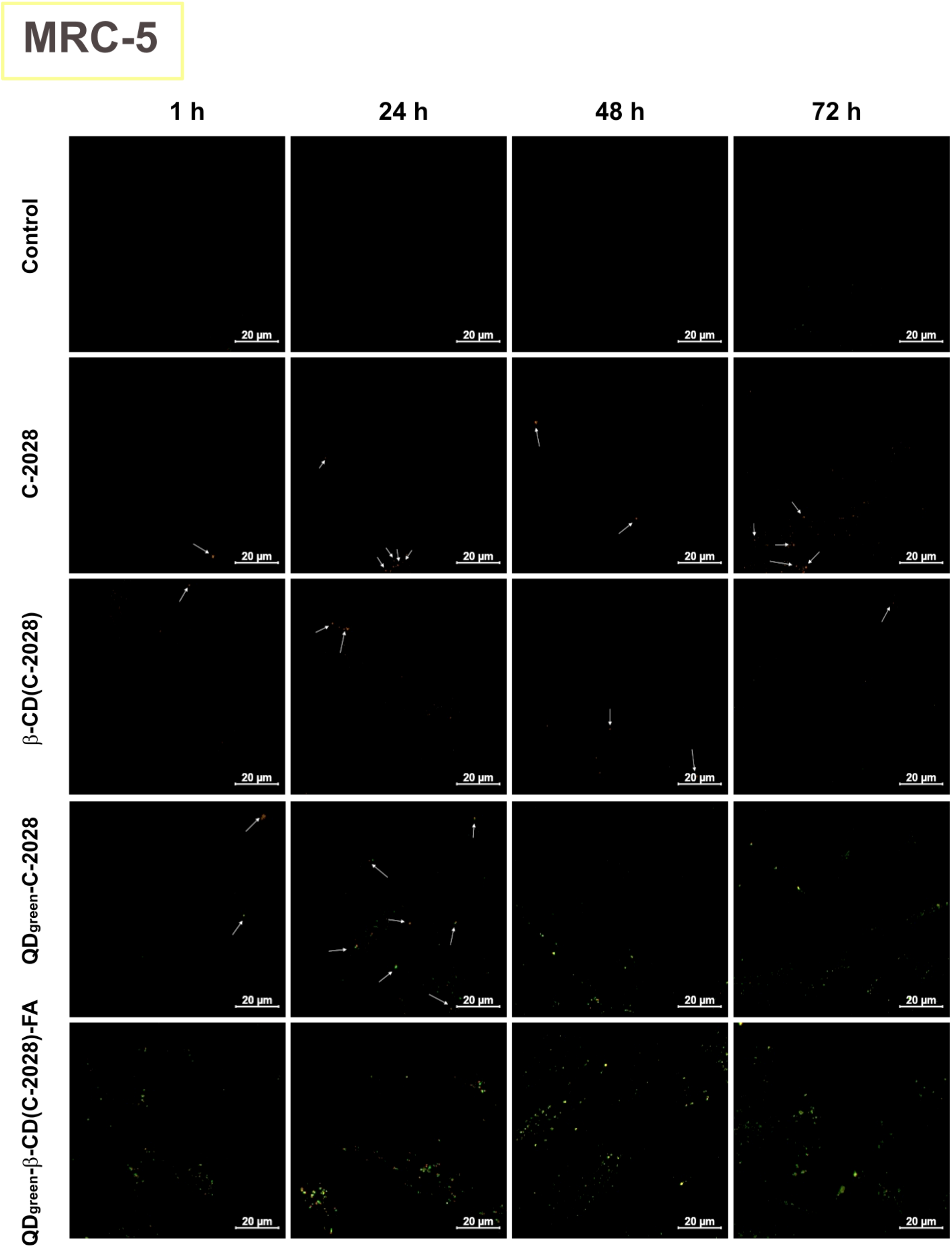
MRC-5
1 h
24 h
48 h
72 h
Control
C-2028
β-CD(C-2028)
QDgreen-C-2028
QDgreen-β-CD(C-2028)-FA
20 μm

**E**

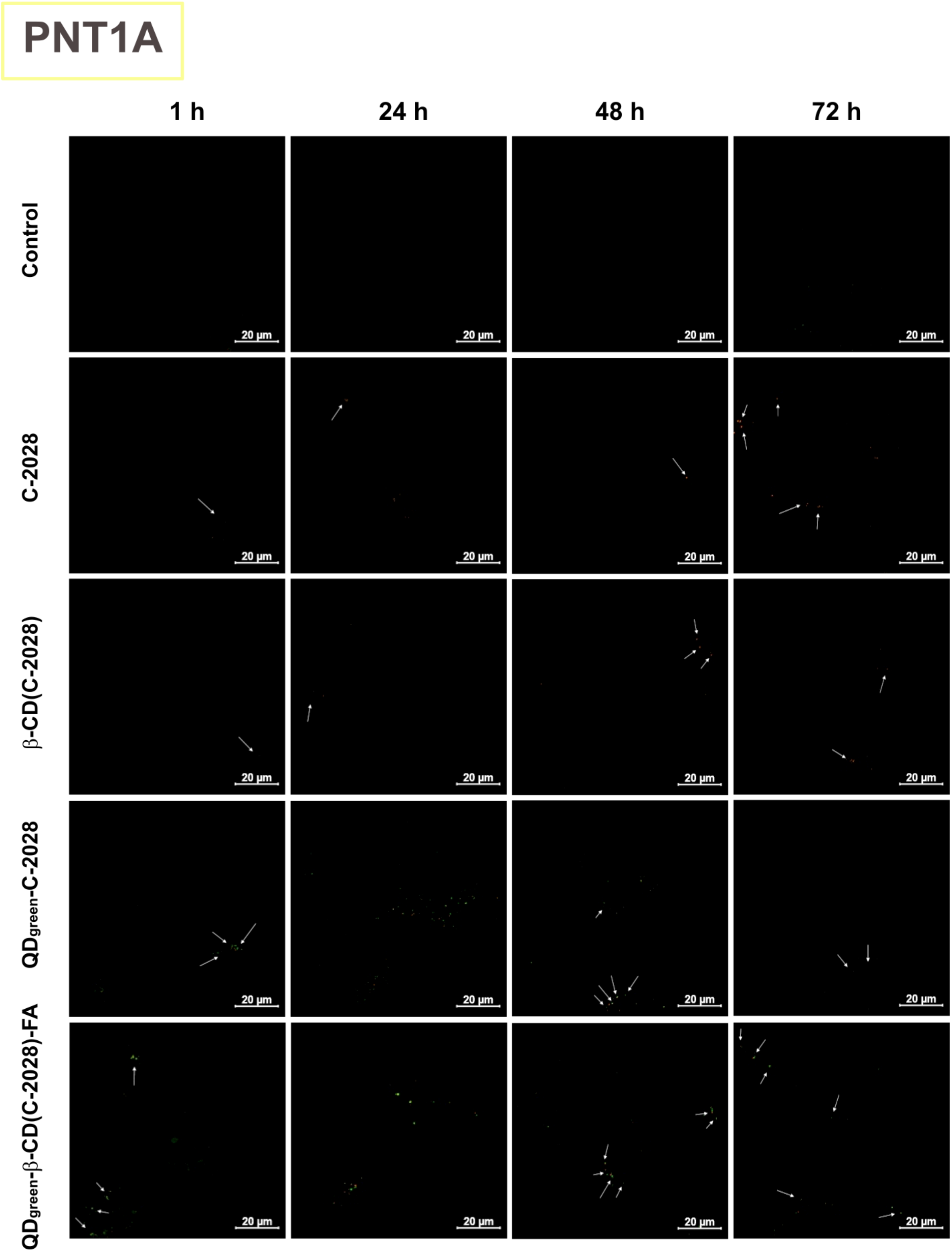


**Figure S4.** Cellular uptake of C-2028, *β*–CD(C-2028), $QD_{green}$-C-2028, and $QD_{green}$-*β*–CD(C–2028)–FA nanoconjugates to cancer (**A**) H460, (**B**) Du-145, (**C**) LNCaP as well as normal (**D**) MRC-5 and (**E**) PNT1A cells for the time indicated and analyzed by CLSM. The scale bar is 20 µm. Data represented the images of three independent experiments.
